# Increased Risk for Severe Malaria in HIV-1–infected Adults, Zambia

**DOI:** 10.3201/eid1505.081009

**Published:** 2009-05

**Authors:** Victor Chalwe, Jean-Pierre Van geertruyden, Doreen Mukwamataba, Joris Menten, John Kamalamba, Modest Mulenga, Umberto D’Alessandro

**Affiliations:** Tropical Diseases Research Centre, Ndola, Zambia (V. Chalwe, D. Mukwamataba, M. Mulenga); Institute of Tropical Medicine, Antwerp, Belgium (J.-P. Van geertruyden, J. Menten, U. D’Alessandro); Thomson Hospital, Luanshya, Zambia (J. Kamalamba)

**Keywords:** HIV/AIDS and other retroviruses, malaria, HIV-1, opportunistic infections, epidemiology, Africa, Zambia, case-control, CME, research

## Abstract

To determine whether HIV-1 infection and HIV-1–related immunosuppression were risk factors for severe malaria in adults with some immunity to malaria, we conducted a case–control study in Luanshya, Zambia, during December 2005–March 2007. For each case-patient with severe malaria, we selected 2 matched controls (an adult with uncomplicated malaria and an adult without signs of disease). HIV-1 infection was present in 93% of case-patients, in 52% of controls with uncomplicated malaria, and in 45% of asymptomatic controls. HIV-1 infection was a highly significant risk factor for adults with severe malaria compared with controls with uncomplicated malaria (odds ratio [OR] 12.6, 95% confidence interval [CI] 2.0–78.8, p = 0.0005) and asymptomatic controls (OR 16.6, 95% CI 2.5–111.5, p = 0.0005). Persons with severe malaria were more likely to have a CD4 count <350/µL than were asymptomatic controls (OR 23.0, 95% CI 3.35–158.00, p<0.0001).

## Abstract 2

In areas in which malaria and HIV-1 are coendemic, adults with severe malaria who show no clinical signs of immunosuppression are likely to be infected with HIV-1.

The geographic overlap between HIV-1 infection and malaria, particularly in eastern and southern Africa, has caused concern since the 1980s. The degree of interaction between HIV-1 infection and malaria emerged during 1999–2009 and has been extensively reviewed for both nonpregnant and pregnant adult women (*1**,**2*). The effect of HIV-1 on malaria seems to be driven mainly by the incapacity of the immune system to control parasite load, leading to a higher prevalence of infection (*3*), a higher incidence of clinical malaria (*4**,**5*), and a risk for treatment failure (*6*) in immunosuppressed HIV-1 patients.

Reports of HIV-1 infection as a risk factor for hyperparasitemia or severe malaria are few and limited. In urban Burkina Faso, >30% of adults with severe malaria were also infected with HIV-1, whereas HIV-1 prevalence in the general adult population was ≈5%–14% (*7*). In South Africa, in an area of low malaria transmission (<1 case/1000/year), HIV-1 infection was associated with severe falciparum malaria (*8**–**10*). Similarly, in Mumbai, India, where malaria transmission is low, HIV-1 prevalence was higher in persons with severe malaria than in the general population (*11*). Therefore, in areas of low malaria transmission, HIV-1 infection seems to be an important risk factor for severe malaria and death. In areas of high malaria transmission (>1/case/1000/year), the relationship between HIV-1 infection and severe malaria is less well established, with a small hospital-based study in Zimbabwe reporting higher risk for severe malaria and related death in HIV-1–infected adults than in HIV-1–uninfected adults (*12*). In all these studies, either the number of cases was small or HIV-1 testing was not performed, and CD4 cell count was not routinely done. The effect of both HIV-1 infection and malaria is important in several African countries with high malaria endemicity. We report the results of a matched case–control study exploring whether HIV-1 is an important risk factor for severe malaria in adults living Luanshya, Zambia, an area of high malaria transmission (>200/cases/year).

## Methods

### Study Design

We chose a case–control design for this study because severe malaria is relatively rare in areas of stable transmission (*13*). Each adult with severe malaria was matched with 2 controls: 1 adult who had uncomplicated malaria and 1 asymptomatic adult in the community. We matched for major confounding variables: age group (15–19, 20–29, 30–39, and 40–49 years), sex, area of residence, and seasonal variation. Controls were recruited within 4 weeks after identification of case-patients.

### Study Site and Patients

The study was conducted at Thompson Hospital, a government district hospital serving Luanshya district in the Copperbelt province of Zambia. In this district, 99% of the population is at risk for malaria (Figure), and 30% of women attending voluntary counseling and testing at the antenatal care department are infected with HIV-1 (*15*). Luanshya is not a site of sentinel surveillance for HIV, but sentinel surveillance conducted during late 2004 and early 2005 in the adjacent district of Ndola showed an HIV prevalence rate of 30% (*16*). From October 2005 through April 2007, all patients 15–49 years of age who sought treatment for symptoms and signs of febrile illness were screened for falciparum malaria by thick and thin blood smear. Patients in whom severe malaria was diagnosed were enrolled after informed consent was given by the patient or by his or her legally authorized representative. We defined severe malaria (*16*) as fever (body temperature >37.5°C ) or history of fever in the previous 48 hours, *Plasmodium falciparum* monoinfection with a density of at least 100 parasites per 200 leukocytes (assumed to be >4,000 parasites/µL) in the absence of any other evident causes of illness, and at least 1 of the following signs: impaired consciousness (Glasgow Coma Score <10), multiple grand mal convulsions, jaundice, hypoglycemia (glycemia <2.5 mmol/L), hyperparasitemia (parasite density >100,000/µL), renal impairment, and cardiorespiratory distress. We collected information about anemia but did not include anemia in the case definition because it can be HIV-1 related, resulting in possible bias, and because uncomplicated and severe malaria episodes can be misclassified in HIV-1–infected persons.

**Figure:**
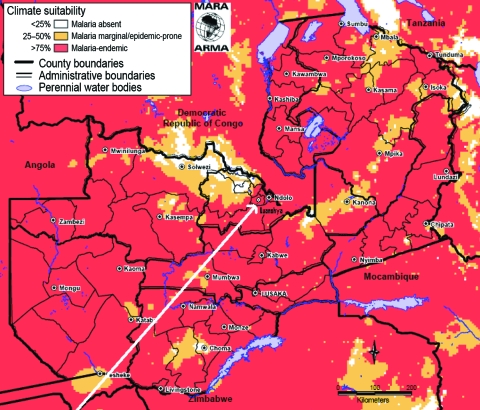
Malaria endemicity in Zambia. This map is a product of the Mapping Malaria Risk in Africa/Atlas du Risque de la Malaria en Afrique (MARA/ARMA) collaboration (www.mara.org.za), July 2005. Malaria distribution was obtained from Craig et al. (*14*). Topographic data were obtained from African Data Sampler, World Resources Institute (www.igc.org/wri/sdis/maps/ads/ads_idx.htm).

Patients with severe malaria were treated according to the national guidelines for severe malaria in Zambia (i.e., quinine 10 mg/kg/8 h for 7 d), with adequate supportive treatment (*17*). On the basis of information about areas of unstable malaria and of HIV surveillance data in Zambia, we predicted that HIV-1 increased the risk for severe malaria 5-fold and assumed a 30% HIV-1 prevalence (primary exposure) among controls. Using a 1:1 case:control ratio, we determined that we needed to recruit 30 case–control pairs to detect this risk with 80% power. Recruitment of cases continued until the required sample size was reached.

Controls with uncomplicated malaria were selected from the clinic closest to the homes of the case-patients. The first patient attending the clinic and exhibiting fever or having a history of fever in the previous 48 hours, without any other disease, with a positive rapid diagnostic test, residing in the catchment area of the health center, matching sex and age group of the case-patient, and willing to participate was recruited as a control. Pregnant women were excluded.

Field trips were organized to case-patients’ residences, where, following the random sampling method, a community control was identified and independently assessed. The first healthy asymptomatic adult fulfilling the matching criteria and willing to participate was recruited as a community control. Pregnant women were excluded. All community controls were assessed for possible confounding factors and screened for falciparum malaria by thick and thin blood smear. We sought to identify controls within 4 weeks after we recruited a case-patient.

### Laboratory Tests

Laboratory technicians were blinded to patient identity and to patient-related parameters. We used microscopy to screen case-patients and community controls for malaria using Giemsa staining 10% for 5 min. A thin blood film was examined to identify type of parasite; a thick film was taken for quantification. Computation of parasite density took into consideration the actual leukocyte count. In asymptomatic controls, we screened for *P. falciparum* infection by using a rapid diagnostic test, Malaria Pf immuno-chromatographic test (ICT) (ICT Diagnostics, Cape Town, South Africa). For each participant, a blood sample (0.5 mL) was collected for HIV-1 testing and CD4 cell count, and for hemoglobin (1 mL), by venipuncture. Neither the study staff nor the patient had access to the HIV-1 test results. HIV-1 testing followed an unlinked anonymous procedure: blood samples were sent to the hospital laboratory, where they were processed anonymously. Determine HIV1/2/O (Abbott Laboratories, Abbott Park, IL, USA) was the first test. If test results were negative, the patient was considered not infected. If results were positive or indeterminate, the blood sample was tested with Unigold Recombinant HIV-1/2 (Trinity Biotech PLC, Bray, Ireland); if results were positive, the patient was classified as HIV-1 infected. If results were negative, the outcome was considered indeterminate, and the sample was further tested with ELISA and Western blot. Patients, after recovering from malaria, and controls were counseled about HIV-1 and offered the opportunity for voluntary counseling and testing.

CD4 counts were determined by flow cytometry on a CyFlow (Cyflow Counter, Partec, Germany) within 5 hours after collection, and multiset software was used to obtain the absolute counts and the CD4+ lymphocyte ratio (*18*). A FACSCount machine (Becton Dickinson, Sparks, MD, USA) was used as a quality control to validate the accuracy of the Cyflow data over time and served as backup.

### Ethics and Consent

The trial was approved by the ethical committee of the Institute of Tropical Medicine, Antwerp, Belgium, and by the Research Ethics Committee of the Tropical Disease Research Centre, Ndola, Zambia. The written informed consent signed by study participants or their legally authorized representatives described the purpose of the study, procedures followed, and risks and benefits of participation. The consent form was in both English and the local language. Patients were counseled on HIV and offered the opportunity to undergo voluntary counseling and testing in conjunction with the study; this procedure was followed to ensure adherence to the standard of counseling and testing stipulated in the guidelines for HIV/AIDS counseling in Zambia (*17*).

### Statistical Analysis

Crude associations between the primary outcome measures and potential risk factors, including HIV-1 status and HIV-1–related immunosuppression (HIV-1+ with CD4 >350/µL and HIV-1+ with CD4 <350/µL), were described by using summary statistics, means, and count and were tested by using Wilcoxon signed rank test for matched variables.

## Results

From December 2005 through March 2007, we recruited 30 case-patients with severe malaria, 30 controls with uncomplicated malaria, and 30 asymptomatic community controls who fulfilled all inclusion criteria. Because of the low malaria incidence among adults, 5 controls with uncomplicated malaria who met the matching criteria could not be identified within 28 days after admission of the case-patient to the hospital. One case-patient and corresponding matched controls were omitted because of missing laboratory results. One case-patient was recruited during March–October 2006, the low malaria transmission season. Median age was 33 years, and men and women were equally represented (Table 1). Eighty-six percent of participants lived in concrete houses. Households contained an average of 4.7 inhabitants, few of whom slept under a bed net (1.5 persons per household, 32%). Only 24% of case-patients (7/29) had slept under a bed net before admission.

**Table 1:** Risk factors for severe malaria in case–control study, Luanshya, Zambia

Risk factors	Case-patients, n = 29	Controls with uncomplicated malaria, n = 29	Asymptomatic controls, n = 29	p value
Demographic characteristics*				
GM† age, y (range)	33 (18–50)	33 (20–49)	33 (18–50)	
No. (%) male	14 (48)	14 (48)	14 (48)	
Living conditions†				
No. (%) living in mud/clay hut	4 (14)	4 (14)	5 (17)	0.91
No. (%) living in concrete house	25 (86)	25 (86)	24 (83)	
GM no. persons living in house* (95% CI)	4.7 (3.7–6.0)	3.6 (2.9–4.5)	4.7 (3.8–5.8)	0.76
Mean no. sleeping under bed net (95% CI)	1.5 (0.7–2.3)	1.5 (0.7–2.3)	1.4 (0.5–2.3)	0.85
No. (%) sleeping under bed net	7 (24)	9 (31)	9 (31)	0.80
No. (%) using antimalarial drug during previous week	13 (45)	8 (28)	5 (17)	0.13
No. using quinine	2	2	1	
No. using sulfadoxine pyrimethamine	8	5	3	
No. using artemether–lumefantrine	5	1	0	
No. (%) with HIV-1	27 (93)	15 (52)	13 (45)	0.03
GM CD4 count (95% CI)‡	173 (125–240)	205 (112–377)	677 (427–1074)	
No. (%) CD4 count <200/µL‡	11/23 (48)	5/14 (36)	1/12 (8)	<0.001
No. (%) CD4 count <350/µL‡	19/23 (83)	11/14 (79)	1/12 (8)	<0.001

*Matching variables. †GM, geometric mean; CI, confidence interval. ‡Not measured in 4 case-patients, 1 control with uncomplicated malaria, and 1 asymptomatic control because of technical constraints.

Impaired consciousness and hypoglycemia were the most common signs of severe malaria in HIV-1–infected case-patients (Table 2). Nineteen percent of patients, all HIV-1 infected, had anemia. All parasite densities were above the fever threshold (2,088/µL–635,500/µL), conservatively set at 2,000 parasites/µL. Seven of 13 patients with a parasite density below the geometric mean were treated with quinine only; others conservatively received some antimicrobial drugs. Six case-patients, all HIV-1–infected, had hyperparasitemia, 5 with >200,000 parasites/µL. None of the case-patients were receiving antiretroviral drugs or cotrimoxazole prophylaxis. Fifteen case-patients received concomitant antimicrobial drug treatment(s) determined by clinical symptoms: penicillin (8 patients), amoxicillin (1), gentamicin (2), metronidazole (1), fluconazole (2), chloramphenicol (3), ciprofloxacin (1), and cotrimoxazole (4). Clinical history, symptoms, outcome, illness duration, and other relevant parameters were similar in case-patients who received concomitant antmicrobial drugs and in those who received quinine only. Five (19%) patients died, 4 within 2 days after admission. Median length of hospital stay for successfully treated patients was 5.5 days. Case-patients were more likely to have used antimalarial treatment (45%) during the week before admission than were controls with uncomplicated malaria (28%) and asymptomatic controls (17%) (Table 1). Three case-patients used antimicrobial agents the week before admission, compared with 2 controls with uncomplicated malaria and 1 asymptomatic control.

**Table 2:** Clinical features of case-patients with severe malaria in case–control study, by HIV-1 infection status, Luanshya, Zambia

Clinical features	HIV-1 infected, n = 27	HIV-1 uninfected,* n = 2
Signs and symptoms, no. (%)		
Fever (>37.5°C)	20 (74)	1 (50)
History of fever	25 (93)	2 (100)
Impaired consciousness (Glasgow Coma Score <10)	15 (56)	0 (0)
Severe anemia (<7 g/dL)†	5 (19)	0 (0)
Convulsions	6 (22)	0 (0)
Jaundice	3 (11)	1 (50)
Hypoglycemia (<2.5 mmol/L)	11 (41)	0 (0)
Hyperparasitemia (>100,000 parasites/µL)	6 (22)	0 (0)
Renal impairment	0 (0.0)	0 (0)
Leukocyte count, mean 1,000/µL (SD)‡	6.9 (3.9)	4.2 and 8.7
Lymphocytes, % (SD)‡	25 (11)	18
Monocytes, % (SD)‡	11 (6)	5.2
Granulocytes, % (SD)‡	62 (20)	76.8
Parasite density, geometric mean/UL (95% confidence interval)	43,314 (25,467–81,145)	11,745 and 38,942
Concomitant antimicrobial drugs	15 (56)	0
Outcome		
No. (%) discharged	24 (82)	2 (100)
Median time hospitalized, d (range)	5.5 (1–31)	4 and 17
No. deceased (case-fatality ratio)	5 (19)	0
Median length of illness before death, d (range)	2 (1–11)	–

*Because only 2 results were HIV negative, no SD or confidence interval was specified. †Anemia was not an inclusion criterion. ‡Not measured in 4 patients because of technical constraints.

### Risk Analysis

Case-patients differed from controls in use of other drugs during the previous week, HIV-1 infection, and CD4 count. No parasitemia was detected in any asymptomatic controls. Because of the matching, controls with uncomplicated malaria and asymptomatic controls were similar to case-patients for all other assessed risk factors (Table 1).

HIV-1 infection was detected in 45% of asymptomatic controls, 52% of controls with uncomplicated malaria, and 93% of case-patients. HIV-1 infection was not a risk factor for uncomplicated malaria (odds ratio [OR] 1.3, 95% confidence interval [CI] 0.5–3.7, p = 0.59) (Table 3). Case-patients were more likely to be infected with HIV-1 than were controls with uncomplicated malaria and asymptomatic controls (OR 12.6, 95% CI 2.0–78.8, p = 0.0005, and OR 16.6, 95% CI 2.5–111.8, p = 0.0005, respectively).

**Table 3:** HIV-1 infection and HIV-1–related immunosuppression as risk factors for nonsevere and severe malaria, case–control study, Luanshya, Zambia*

Participant characteristics	No. (%)	OR (95% CI)	p value	OR (95% CI)	p value
HIV-1 infected†					
Asymptomatic controls	13/29 (45)	1	–		
Controls with uncomplicated malaria	15/29 (52)	1.3 (0.5–3.7)	0.59	1	–
Case-patients (severe malaria)	27/29 (93)	16.6 (2.5–111.8)	0.0005	12.6 (2.0–78.8)	0.0005
CD4 cell count <350/µL‡					
Asymptomatic controls	1/12 (8)	1	–		
Controls with uncomplicated malaria	11/14 (79)	7. 67 (1.78–33.01)	0.001	1	–
Case-patients (severe malaria)	19/23 (83)	23.00 (3.35–158.00)	<0.0001	3.00 (0.83–10.83)	0.08

*OR, odds ratio; CI, confidence interval. †p value obtained by using matched-pairs signed-ranks test. ‡CD4 count not measured in 4 HIV-1–infected case-patients, 1 control with uncomplicated malaria, and 1 asymptomatic control because of technical constraints. p value obtained by using Wilcoxon signed-ranks test and Wilcoxon rank-sum test.

Eighty-three percent of case-patients had a CD4 count <350/µL, compared with 79% of controls with uncomplicated malaria (p = 0.76) and 8% of asymptomatic controls (p<0.0001). Controls with uncomplicated malaria were more likely than asymptomatic controls to have a CD4 count <350/µL (OR 7.67, 95% CI 1.78–33.01, p = 0.001). Case-patients were more likely to have a CD4 count <350/µL than were asymptomatic controls (OR 23.00, 95% CI 3.35–158.00, p<0.0001) but not controls with uncomplicated malaria (OR 3.00, 95% CI 0.83–10.83, p = 0.32).

The extremely high proportion of low CD4 count in both case-patients and controls with uncomplicated malaria might be confounded by a temporary malaria-induced reallocation of specific T-cells (*19*). Therefore, 28 days after successful treatment, the absolute CD4 count was measured in 10 HIV-infected case-patients. During this period, the mean CD4 count increased >2-fold, from 142 (95% CI 76–269) to 320 CD4/µL (95% CI 169–607) (data not shown). However, the proportion of case-patients with CD4 count <350/µL remained substantial (70%), although slightly less than at admission (90%).

## Discussion

In Luanshya, Zambia, an area where malaria is mesoendemic, HIV-1 infection is an important risk factor for severe malaria in adults, primarily in those with a low CD4 count. The increased risk for severe malaria in HIV-1–infected persons already has been reported from areas of low and unstable transmission (*8**–**11*) but never has been firmly established in areas of stable malaria transmission. The paucity of information results from the difficulty of obtaining it. Studies collecting relevant information retrospectively are vulnerable to considerable bias, and prospective studies are difficult to carry out where adults have acquired immunity and severe malaria is rare (*20*). In our study, recruitment of 30 persons with severe malaria from a population of 10,000 persons took 2 years. However, even prospectively, diagnosing severe malaria with certainty might be difficult, particularly in the presence of HIV-1 co-infection, because several opportunistic infections of AIDS patients could have clinical presentations similar to those of severe malaria. Persons with severe malaria could have had a concomitant *Streptococcus pneumoniae* or *Salmonella* *enterica* serovar Typhimurium infection (*21*) or HIV-1 immunosuppression-related meningitis, such as cryptococcal meningitis. Although, none of the case-patients we recruited were identified by the treating physicians as having clinical AIDS, in the absence of a blood culture, physicians conservatively decided to concomitantly prescribe antimicrobial drugs in 15 cases. More detailed review of patients’ files showed that most of these persons had hypoglycemia or hyperparasitemia, and none were in septic shock. Moreover, these patients recovered rapidly after treatment, and all but 1 were recruited during the high malaria transmission season, which support the argument that these persons had true cases of severe malaria.

The increased risk for uncomplicated malaria in HIV-1–infected patients with a low CD4 count is consistent with information in several cohort studies (*1*). Although the extremely high proportion of a low CD4 count in patients in our study might have been confounded by a temporary malaria-induced reallocation of specific T cells; 70% had a CD4 count <350/µL 1 month after successful treatment. Therefore, immunosuppression is likely an additional risk, but absolute CD4 count cannot be interpreted during a severe malaria episode.

Almost half of malaria cases throughout the world occur in areas where the disease is holoendemic (*22*). HIV-1 program managers working in areas where both diseases are prevalent should be aware that HIV-1 infection—and certainly HIV-1–related immunosuppression—are important risk factors for severe malaria. Early detection of HIV-1 infection is extremely important because comprehensive measures to prevent malaria and chemoprophylaxis with cotrimoxazole could be promptly implemented to protect against uncomplicated and severe malaria, a disease with a high fatality rate (*23*). Clinicians in such settings also should be aware of the strong association between severe malaria and HIV-1 so they can assess patients for other underlying diseases and offer the opportunity for voluntary counseling and testing for HIV-1 when patients have recovered from malaria.

## References

[1] Hewitt K, Steketee R, Mwapasa V, Whitworth J, French N. Interactions between HIV and malaria in non-pregnant adults: evidence and implications. AIDS. 2006;20:1993–2004. 10.1097/01.aids.0000247572.95880.92 17053345

[2] ter Kuile FO, Parise ME, Verhoeff FH, Udhaya KV, Newman RD, van Eijk AM, The burden of co-infection with human immunodeficiency virus type 1 and malaria in pregnant women in sub-Saharan Africa. Am J Trop Med Hyg. 2004;71:41–54. 15331818

[3] Whitworth J, Morgan D, Quigley M, Smith A, Mayanja B, Eotu H, Effect of HIV-1 and increasing immunosuppression on malaria parasitaemia and clinical episodes in adults in rural Uganda: a cohort study. Lancet. 2000;356:1051–6. 10.1016/S0140-6736(00)02727-6 11009139

[4] French N, Nakiyingi J, Lugada E, Watera C, Whitworth JA, Gilks CF. Increasing rates of malarial fever with deteriorating immune status in HIV-1-infected Ugandan adults. AIDS. 2001;15:899–906. 10.1097/00002030-200105040-00010 11399962

[5] Patnaik P, Jere CS, Miller WC, Hoffman IF, Wirima J, Pendame R, Effects of HIV-1 serostatus, HIV-1 RNA concentration, and CD4 cell count on the incidence of malaria infection in a cohort of adults in rural Malawi. J Infect Dis. 2005;192:984–91. 10.1086/432730 16107950

[6] Van geertruyden JP, Mulenga M, Mwananyanda L, Chalwe V, Moerman F, Chilengi R, et al. HIV-1 immune suppression and antimalarial treatment outcome in Zambian adults with uncomplicated malaria. J Infect Dis. 2006;194:917–25. 10.1086/507310 16960779

[7] Diallo AH, Ki-Zerbo G, Sawadogo AB, Guiguemde TR. Severe malaria and HIV in adult patients in Bobo-Dioulasso, Burkina Faso [article in French]. Med Trop (Mars). 2004;64:345–50. 15615384

[8] Grimwade K, French N, Mbatha DD, Zungu DD, Dedicoat M, Gilks CF. Childhood malaria in a region of unstable transmission and high human immunodeficiency virus prevalence. Pediatr Infect Dis J. 2003;22:1057–63. 10.1097/01.inf.0000101188.95433.60 14688565

[9] Grimwade K, French N, Mbatha DD, Zungu DD, Dedicoat M, Gilks CF. HIV infection as a cofactor for severe falciparum malaria in adults living in a region of unstable malaria transmission in South Africa. AIDS. 2004;18:547–54. 10.1097/00002030-200402200-00023 15090809

[10] Cohen C, Karstaedt A, Frean J, Thomas J, Govender N, Prentice E, Increased prevalence of severe malaria in HIV-infected adults in South Africa. Clin Infect Dis. 2005;41:1631–7. 10.1086/498023 16267737

[11] Khasnis AA, Karnad DR. Human immunodeficiency virus type 1 infection in patients with severe falciparum malaria in urban India. J Postgrad Med. 2003;49:114–7. 12867684

[12] Chirenda J, Siziya S, Tshimanga M. Association of HIV infection with the development of severe and complicated malaria cases at a rural hospital in Zimbabwe. Cent Afr J Med. 2000;46:5–9. 14674199 10.4314/cajm.v46i1.8514

[13] Hayes RJ, Marsh K, Snow RW. Case-control studies of severe malaria. J Trop Med Hyg. 1992;95:157–66. 1597871

[14] Craig MH, Snow RW, le Sueur D. A climate-based distribution model of malaria transmission in sub-Saharan Africa. Parasitol Today. 1999;15:105–11. 10.1016/S0169-4758(99)01396-4 10322323

[15] Central Board of Health, Ministry of Health, Government of Republic of Zambia (GRZ). Zambia 2004 antenatal clinic sentinel surveillance. Lusaka (Zambia): GRZ; 2007.

[16] Severe falciparum malaria. World Health Organization, communicable diseases cluster. Trans R Soc Trop Med Hyg. 2000;94(Suppl 1):S1–90. 11103309

[17] Central Board of Health, Ministry of Health, Government of Republic of Zambia (GRZ). Zambia. Management of antiretroviral therapy. A reference manual for health workers. Lusaka (Zambia): GRZ; 2004.

[18] Janossy G, Jani I, Göhde W. Affordable CD4(+) T-cell counts on ‘single-platform’ flow cytometers I. Primary CD4 gating. Br J Haematol. 2000;111:1198–208. 10.1046/j.1365-2141.2000.02433.x 11167762

[19] Van geertruyden JP, Mulenga M, Kasongo W, Polman K, Colebunders R, Kestens L, et al. CD4 T-cell count and HIV-1 infection in adults with uncomplicated malaria. J Acquir Immune Defic Syndr. 2006;43:363–7. 10.1097/01.qai.0000243125.98024.da 17079994

[20] Butcher GA. T-cell depletion and immunity to malaria in HIV-infections. Parasitology. 2005;130:141–50. 10.1017/S003118200400650X 15727063

[21] Gilks CF, Brindle RJ, Otieno LS, Simano PM, Newnham RS, Bhatt SM, Life-threatening bacteraemia in HIV-1 seropositive adults admitted to hospital in Nairobi, Kenya. Lancet. 1990;336:545–9. 10.1016/0140-6736(90)92096-Z 1975046

[22] Hay SL, Guerra CA, Tatem AJ, Noor AM, Snow RW. The global distribution and population at risk of malaria: past, present, and future. Lancet Infect Dis. 2004;4:327–36. 10.1016/S1473-3099(04)01043-6 15172341 PMC3145123

[23] Mermin J, Ekwaru JP, Liechty CA, Were W, Downing R, Ransom R, Effect of co-trimoxazole prophylaxis, antiretroviral therapy, and insecticide-treated bednets on the frequency of malaria in HIV-1-infected adults in Uganda: a prospective cohort study. Lancet. 2006;367:1256–61. 10.1016/S0140-6736(06)68541-3 16631881

